# Effects of nonalcoholic fatty liver disease on sarcopenia: evidence from genetic methods

**DOI:** 10.1038/s41598-024-53112-1

**Published:** 2024-02-01

**Authors:** Jiaqin Yuan, Jinglin Zhang, Qiang Luo, Lipeng Peng

**Affiliations:** 1 Department of Orthopedics, The Second People’s Hospital of Yibin, Sichuan, China; 2 Department of Occupational Diseases, Yibin Center for Disease Control and Prevention, Sichuan, China; 3 Department of Cardiology, Children’s Hospital of Chongqing Medical University, Chongqing, China

**Keywords:** Medical genetics, Non-alcoholic fatty liver disease

## Abstract

With the aging of the population, sarcopenia has become more common. Studies have shown a broad association between liver disease and sarcopenia. However, this link remains unclear. Our study explored the link between NAFLD and sarcopenia and predicting the pathogenesis. To begin, we investigated the causal relationship and genetic correlation between them using MR and LDSC. Second, each GWAS was annotated by MAGMA. The annotated genes were analyzed for pleiotropy using the PLACO approach. Finally, functional analysis was conducted on the identified pleiotropic genes. We observed a significant genetic correlation between NAFLD and sarcopenia. Subsequently, we conducted gene-level pleiotropy analysis using PLACO and identified a total of 153 genes with pleiotropic effects. Functional analysis revealed enrichment of these genes in various tissues, including pancreas, liver, heart, blood, brain, and muscle, with involvement in cellular regulation, intracellular function, and antigen response. Moreover, our MR analysis provided evidence of a causal relationship between NAFLD and sarcopenia. Our study has discovered the genetic and causal relationships between NAFLD and sarcopenia, providing further insights into their pathophysiological mechanisms. The identification of pleiotropic genes also offers potential targets for future drug therapies aimed at controlling or treating NAFLD and sarcopenia.

## Introduction

Sarcopenia is a term first introduced in the late 1980s by Dr. I.H. Rosenberg^1^. The term is of Greek origin and means muscle loss (sarcos = flesh, penia = lack). It used to be considered an aging-related syndrome, but with more research, it is now considered a progressive disease associated with metabolic syndrome, liver disease, and cardiovascular disease^2,3^. The concept of sarcopenia was updated by the European Sarcopenia Working Group in 2018^4^. It defined sarcopenia by low measurements in three aspects: Muscle strength, muscle amount/quality and physical function, and considered it as a progressive and systemic skeletal muscle disease that can cause further negative consequences such as falls, fractures, disability, and death.

Non-alcoholic fatty liver disease (NAFLD) is a clinicopathologic syndrome characterized by excessive fat deposition in hepatocytes without the presence of alcohol or other apparent hepato-protective factors^5^. It is an acquired metabolic stress liver injury closely associated with insulin resistance and genetic susceptibility and encompasses a spectrum of diseases, including simple fatty liver (NAFL), non-alcoholic steatohepatitis (NASH), and associated cirrhosis. According to 2016 data, the prevalence of NAFLD was 25.24% worldwide and is steadily increasing^6^.

Considering that both NAFLD and sarcopenia are closely related to the body's metabolic factors, the question of whether there is a correlation between the two has attracted the attention of many scholars. Numerous observational studies have demonstrated an association between sarcopenia and chronic liver disease^7–9^. Takahashi et al. reported sarcopenia was an independent risk factor for nonalcoholic steatohepatitis (NASH) and NAFLD with severe fibrosis. And, Lee et al. also found sarcopenia has been independently related to an increased risk of NAFLD and advanced fibrosis^9,10^. However, the results of observational studies may be influenced by confounding factors or systematic biases, e.g., reverse causality, measurement bias, etc. Thus, Mendelian randomization (MR) was used to assess causality, which limits (rather than completely eliminates) bias due to confounding and reverse causality.

According to the EWGSOP working group, low muscle strength is the most important indicator of sarcopenia^4^. Sarcopenia is severe when there is low muscle strength, low muscle mass, and low physical function. Therefore, hand strength (GS) and appendicular lean mass (ALM) were used as the primary indicators and bioelectrical impedance (BIA) as a secondary indicator in our study.

First, we employed MR to assess the causal relationship between NAFLD and sarcopenia (primary and secondary indicators). In addition, we quantified the extent to which NAFLD and sarcopenia (primary indicator) share a common genetic basis by applying the cross-trait linkage disequilibrium score regression (LDSC) approach^11^. Finally, a gene-centric pleiotropy analysis was performed (primary indicator). Multi-marker Analysis of Genomic Annotation (MAGMA)^12^ was applied to aggregate SNP-level association signals to individual gene-level association signals, and PLACO analysis^13^ was performed based on this.

## Methods

### Data source

A meta-analysis of eight cohorts in genome-wide association studies (GWAS) provided summary genetic data for non-alcoholic fatty liver disease (NAFLD)^14^. The meta-analysis included 377,998 cases of European ancestry, including 4,761 NAFLD cases, and 373,227 controls. NAFLD patients were those who had one of the following ICD codes: ICD-10 K75.8 for "nonalcoholic fatty liver disease," K76.0 for "nonalcoholic steatohepatitis," or ICD-9 571.5 for "nonalcoholic fatty liver disease." Additional details on the research design, which include aspects such as sample collection, quality assurance protocols, and computational techniques, can be found in the primary publication and are listed in Table S1.

In the present study, there were three assessment indicators of sarcopenia: grip strength and appendicular lean mass (primary indicator) and bioelectrical impedance (secondary indicator). For GWAS data on handgrip strength (left and right) and bioelectrical impedance (left and right), we obtained 9,851,867 single nucleotide polymorphism (SNP) markers from the MRC-IEU consortium. Handgrip strength (left) and handgrip impedance (right) were derived from 461,026 and 461,089 European ancestry, respectively. Leg impedance (left) and leg impedance (right) were derived from 454,857 and 454,863 European ancestry, respectively. All of the above data are available on the IEU OpenGWAS project website (https://gwas.mrcieu.ac.uk/).

We obtained summary-level GWAS association data for appendicular lean mass (ALM) through the published study by Pei et al.^15^. Most participants were recruited between the ages of 48 and 73. The final sample included 450,243 individuals, of whom 244,730 were female and 205,515 were male. The total amount of fat-free mass in the arms and legs was used to calculate ALM. For each sex, the original ALM values were adjusted using covariates (appendicular fat mass, age, age squared, genotyping array, etc.).

### MR analysis

In the MR study, genetic variation served as instrumental variables (IV) to assess the causal relationship between risk factors and resulting outcomes^16^. We used single nucleotide polymorphisms (SNPs) with genome-wide significance (P < 5E-06) as instrumental variables and clumping them based on the 1000 Genomes Project linkage disequilibrium structure. We kept index SNPs with minimal *P* values (R2 < 0.001, with all other relevant SNPs within 10,000 kb). For each IV, we first evaluated the percentage of NAFLD phenotypic variation explained (PVE) by the instrumental variable, and we quantitatively assessed the strength of the instruments by calculating the F-statistic, which is generally considered free of bias for weak instrumental variables when the F-statistic is > 10. Next, we conducted a two-sample MR study which primarily used an inverse variance weighting (IVW) random effects method to assess the causal effects of NAFLD on sarcopenia (primary and secondary indicators)^17^. Then, complete the assessment of causal relationships using the maximum likelihood method (ML) and the weighted median method (WM). The IVW method is applied under the assumption that all instrumental variables are valid. Thus, the method contributes to accurate estimation of the results.

To assess the robustness of the outcomes, we also performed a number of sensitivity and pleiotropy assessments. First, we analyzed the heterogeneity of the included instrumental variables using the Cochran Q statistic and the "leave-one-out" (LOO) approach. Second, we used MR Egger regression to account for pleiotropy. Finally, we performed global, outlier, and distortion tests using the MR pleiotropy residual sum and outlier test (MR-PRESSO) method as an additional control for pleiotropy^18^.

### Genetic correlation via LD score regression

We evaluated the common polygenic structure between NAFLD and sarcopenia using the cross-trait linkage disequilibrium score regression (LDSC) approach (denoted r_g_)^11^. LD Scores were calculated based on the European samples from the 1000 Genomes Project as the reference panel^19^. Theoretically, even though the GWAS for NAFLD and sarcopenia overlap, the slope of the regression model of LDSC can offer an objective measure of genetic relationships. Before LDSC, we performed a rigorous quality check for each SNP: 1) All non-biallelic alleles and strand-ambiguous alleles (A/T, C/G) were excluded; 2) alleles with a MAF < 0.01 and no or duplicate rs numbers were excluded; 3) SNPs with mismatched alleles in the 1000 Genomes Project were excluded. To avoid the effects of multiple testing, we used the false discovery rate (FDR) to adjust the P-values for LDSC.

### Gene-centric pleiotropy analysis

To explore pleiotropic genes, we converted summary-level SNPs into gene-level signals of association using the MAGMA method. It has been shown that MAGMA analysis is a powerful gene-based association approach with excellent computational efficiency. First, we defined the set of SNPs as those located within a particular gene using the VEGAS annotation file. Then, the *P* values of the SNPs within each gene are weighted averaged to obtain a gene-level *P* value, and the *P* values are simultaneously transformed into Z-statistics. Finally, the newly determined Z-statistic was subjected to a pleiotropy test using the PLACO method.

PLACO is an innovative method for detecting pleiotropy at the level of SNPs using the concept of composite null hypothesis from high-dimensional mediation analysis^12^. Previous simulations^20^ and variance-component-based mediation analyses under the composite null hypothesis^21^ have suggested the potential use of this method to assess validity at the gene level. Consequently, we used it to identify polymorphic associations at the gene level. To mitigate the impact of excessively large effects, SNPs with extreme Z^2^ (> 80) values were excluded. PLACO assumes three sub-null scenarios for each gene studied using the composite null hypothesis of pleiotropy: (i) H00: The gene is not associated with either disease. (ii) H01: The gene has an effect only on the first disease. (iii) H02: The gene has an effect only on the second disease. (iv) H1: the gene effect on both diseases, which represents a pleiotropic relationship. To avoid the effects of multiple testing, we used the false discovery rate (FDR) to adjust the P-values for both MAGMA and PLACO.

### Functional analysis for pleiotropic genes

We performed differential expression analysis and gene set enrichment analysis with FUMA for the pleiotropic genes analyzed by PLACO^22^. The gene expression dataset is primarily from GTEx v8, which contains 54 different tissue types and includes a total of 15,201 samples^23^. To avoid the effects of multiple testing, we used the false discovery rate (FDR) to adjust the P-values for GTEx V8 analysis. Differentially expressed gene sets (DEG) were pre-calculated by performing a two-tailed t-test for any gene against all others. Prior to this, expression values were normalized using a log2 transformation of expression values. Genes with a Bonferroni-corrected *P* value ≤ 0.05 and an absolute log change ≥ 0.58 were defined as the DEG set for a given tissue. Based on the DEG set, the up- and down-regulated genes were further calculated by considering the sign of the t-statistic.

We also performed a functional enrichment analysis of the identified 24 pleiotropic genes using the online analysis tool DAVID^24^. This analysis included functional Gene Ontology annotations (GO) to prioritize and interpret the functions of these genes. The results were visualized to provide insights into the important roles of these genes.

### Ethical approval and consent to participate

The information utilized for this research can be accessed publicly, was approved ethically, and the participants gave their informed permission.

## Results

### MR analysis results

**Figure 1:**
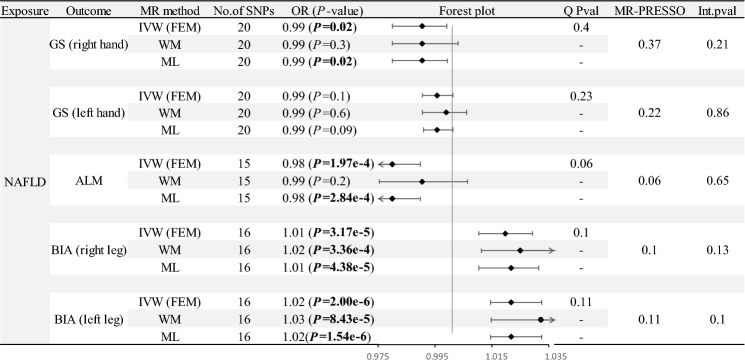
Causal effect of NAFLD on sarcopenia estimated with different MR methods. GS: hand strength; ALM: appendicular lean mass; BIA: bioelectrical impedance.

We included a total of 22 SNPs that met the instrumental variable conditions. However, during the analysis process, we removed some SNPs with heterogeneity and pleiotropy. Table 1 shows the details. The PVE of all IVs was 1.1%. All IVs had an F-statistic greater than 10 (from 21 to 267). The absence of heterogeneity and horizontal pleiotropy is not found in all MR analyses, so we used the fixed effects model (FEM) to estimate causal effects. As a result, we found evidence of a causal relationship between NAFLD and grip strength, BIA (leg), and ALM. Notably, NAFLD demonstrated a slight but statistically significant negative correlation with right-hand grip strength, indicated by an OR of 0.99 (95% CI, 0.98–0.99; P = 0.02). However, this association was not observed in the left hand. In contrast, a positive association was observed between NAFLD and BIA measurements, with both the right and left legs showing similar odds ratios (OR = 1.02 [95% CI, 1.01–1.03]; P-values of 3.17E-5 and 1.54E-6, respectively). Furthermore, NAFLD was negatively associated with ALM, as indicated by an odds ratio of 0.98 (95% CI, 0.97–0.99; P = 1.97E-4). These results are also similar for weighted median analysis and maximum likelihood analysis, as detailed in Fig. 1.

**Table 1:** Characteristics of SNPs included as instrumental variables in MR analysis.

SNP	EA	OA	EAF	Beta	SE	*P* val	*F*-statistic
rs4922548	T	C	0.03	− 0.26	0.06	4.83E-06	21
rs2862954*	C	T	0.50	− 0.09	0.02	3.21E-06	22
rs113358402	A	G	0.01	0.34	0.07	1.53E-06	23
rs4772750	G	C	0.39	0.10	0.02	2.80E-06	22
rs58105980	G	A	0.18	− 0.14	0.03	2.76E-07	26
rs146055091	C	A	0.02	0.28	0.06	2.25E-06	22
rs182611493	G	A	0.01	0.45	0.07	2.33E-11	45
rs73001065*	C	G	0.07	0.35	0.03	1.08E-24	105
rs429358‡#	C	T	0.16	− 0.20	0.03	2.17E-11	45
rs10798888	T	G	0.17	0.14	0.03	4.65E-07	25
rs1760216*‡#	G	T	0.21	0.12	0.03	1.07E-06	24
rs2642442	T	C	0.32	0.14	0.02	7.67E-10	38
rs187266580	A	G	0.02	0.18	0.04	3.94E-06	21
rs3747207*	A	G	0.21	0.37	0.02	6.74E-60	267
rs1260326*‡#	C	T	0.39	− 0.14	0.02	2.54E-11	45
rs112631549	T	C	0.07	0.17	0.04	4.08E-06	21
rs73952150	T	G	0.18	0.12	0.03	3.32E-06	22
rs17671252	T	C	0.23	0.11	0.02	1.07E-06	24
rs147732494	C	G	0.11	0.13	0.03	1.68E-06	23
rs1061187	A	G	0.10	0.15	0.03	1.70E-06	23
rs542004583*	G	A	0.02	0.24	0.05	2.82E-07	26
rs17321515‡#	G	A	0.48	− 0.15	0.02	1.81E-13	54

*Indicates the presence of pleiotropic SNPs that were not included in the MR analysis of NAFLD and ALM.

^‡^Indicates the presence of pleiotropic SNPs that were not included in the MR analysis of NAFLD and BIA (right leg).

^#^Indicates the presence of pleiotropic SNPs that were not included in the MR analysis of NAFLD and BIA (left leg).

EA, effect allele; OA, other allele; EAF, effect allele frequency; SE, standard error.

To validate these associations, we performed sensitivity analyses, including Cochran’s Q test, MR-Egger regression and MR-PRESSO, and found no evidence of heterogeneity of effects across instrumental variables or horizontal pleiotropy (Fig. 1). Finally, LOO analysis shows that no single IV can dominate the causal relationship (Figure S1).

### Estimated genetic correlation

Utilizing LDSC, we estimated SNP-based heritability (ĥ2) for several traits. Notably, the heritability for NAFLD was relatively low at 0.96% (SE = 0.001). In contrast, grip strength presented higher heritability values, with 9.98% (SE = 0.004) for the right hand and 10.27% (SE = 0.004) for the left hand. Remarkably, the highest heritability was observed for ALM, estimated at 40.55% (SE = 0.017). Then, we found a significant positive genetic correlation between NAFLD and two-handed grip strength (right: $\widehat{{\text{r}}}$_g_ = 0.166, P _FDR_ = 2.00 × 10^–4^; left: $\widehat{{\text{r}}}$_g_ = 0.195, P _FDR_ = 3.00 × 10^–5^), ALM ($\widehat{{\text{r}}}$_g_ = 0.175, P_FDR_ = 1.67 × 10^–5^) by cross-trait genetic correlation analysis. This link implies a possible common genetic cause between them. Hence, it is necessary to further investigate the genetic mechanism.

### Shared associated genes

For gene-level pleiotropy analysis, we independently obtained 18,309, 18,414, and 18,207 genes from the GWAS of NAFLD, ALM, and GS, respectively. Subsequently, we used these genes for pleiotropy analysis by PLACO. Therefore, PLACO analysis identified 1,462 statistically significant genes (P_placo_ < 0.05), of which 32 had significant associations (FDR < 0.05) with both NAFLD and GS. This corresponds to the identification of 0.18% of genes with pleiotropic effects. Finally we found that 1,910 genes were statistically significant (P_placo_ < 0.05), of which 153 had significant associations (FDR < 0.05) with both NAFLD and ALM. This accounts for 0.83% of the total number of ALM genes. Note that we found 24 genes present in both the GS and ALM results. Full details can be found in Table S2.

### Gene set functional analysis

**Figure 2:**
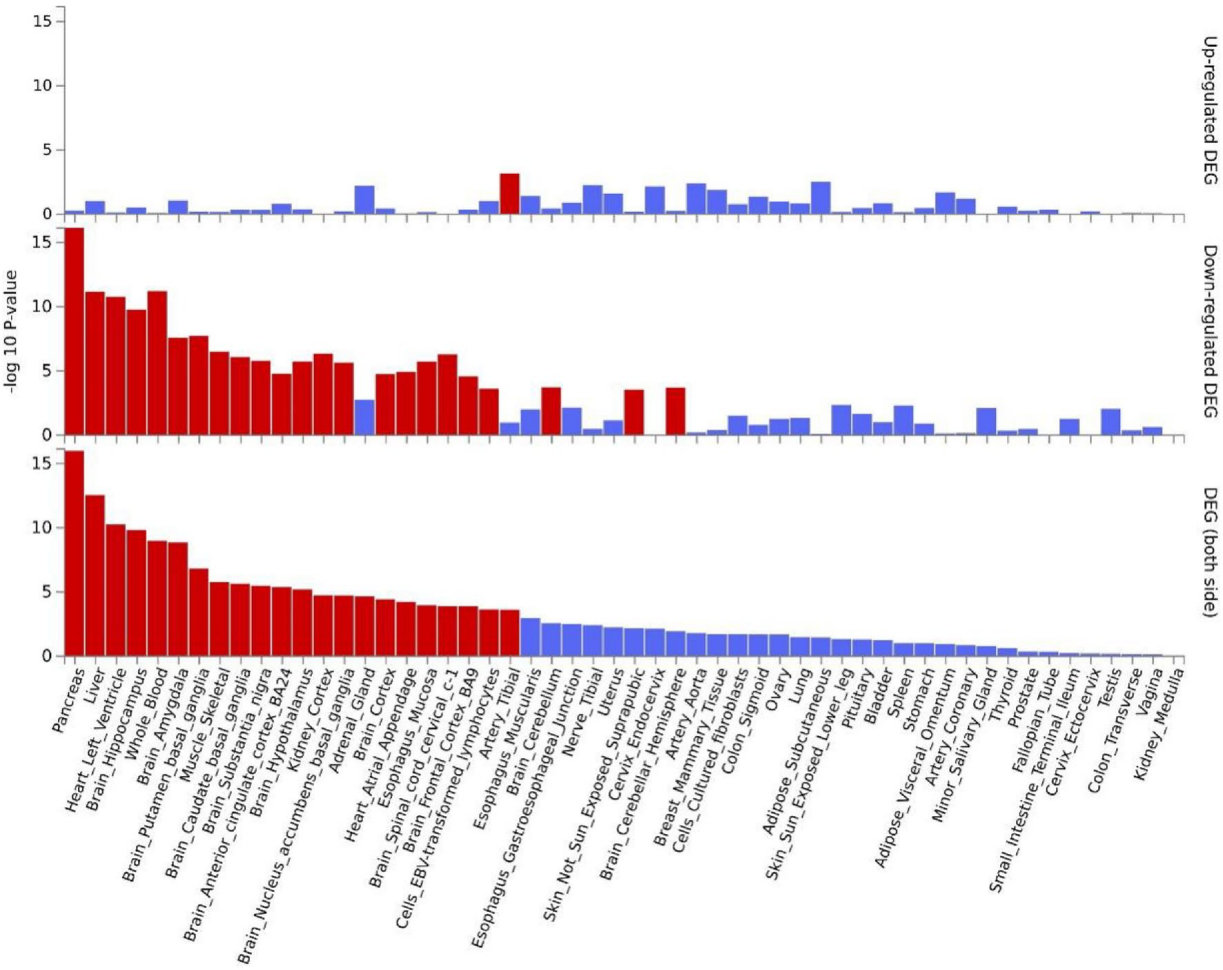
Enrichment of differentially expressed ones of all identified pleiotropic genes based on expression levels in the 54 GTEx v8 project. (*P* values are shown in the yaxis with a scale of -log10. The bars in red represent significant enrichment with Bonferroni adjustment for multiple hypothesis testing).

We also performed gene enrichment analysis using GTEx V8 for the 161 pleiotropic genes identified by PLACO. In GTEx V8, our analysis revealed that genes differentially expressed in these conditions were predominantly enriched in a range of tissues, including the pancreas (P_FDR_ = 1.03 × 10^–15^), liver (P_FDR_ = 2.43 × 10^–11^), heart (P_FDR_ = 1.54 × 10^–9^), blood (P_FDR_ = 3.61 × 10^–8^), brain (P_FDR_ = 3.95 × 10^–8^), and muscle (P_FDR_ = 4.37 × 10^–5^). In addition, the down-regulated differentially expressed genes were also found to be significantly enriched in these tissues (Fig. 2).

**Figure 3:**
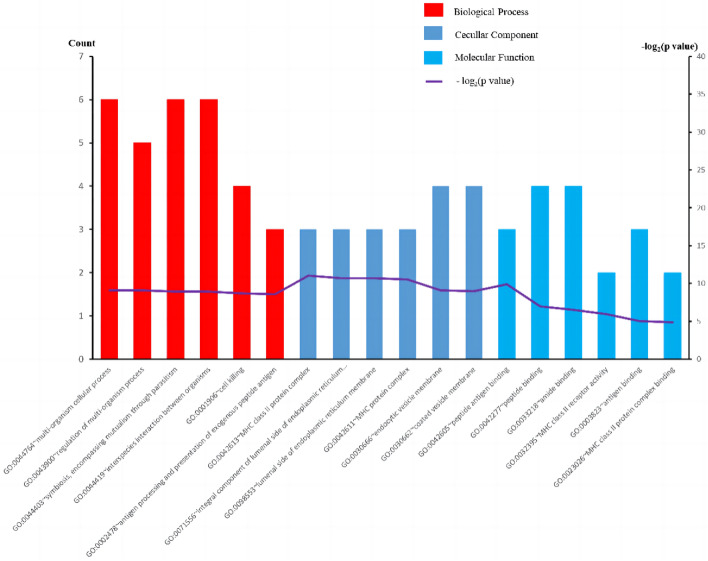
Top 6 significant types of pathways in terms of the GO enrichment analyses. BP: biological process; CC: cellular component; MF: molecular function.

Furthermore, GO analyses were performed specifically for the 24 pleiotropic genes shared between ALM and GS. In addition, we conducted GO analyses for the 24 pleiotropic genes shared between ALM and GS. The results showed an enrichment of biological processes (BP) associated with regulatory and intercellular processes in the organism. In terms of cellular components (CC), these genes showed enrichment in cellular endoplasmic reticulum membranes, MHC protein complexes, and cellular vesicle membranes. As for molecular function (MF), genes were enriched in functions such as peptide-antigen binding and peptide binding. The most representative GO terms are shown in Fig. 3. Enrichment analysis revealed that these genes play critical roles in the regulation of cytokines, biosynthetic processes, and cytokine-mediated signaling pathways, which also supports the validity of genetic pleiotropy.

## Discussions

To our knowledge, this study represents the first systematic assessment to date of the association between NAFLD and sarcopenia, combining LDSC, PLACO pleiotropic analysis, and MR methods. Based on the largest GWAS summary statistics available, LDSC analysis revealed a positive genetic correlation between NAFLD and sarcopenia. Subsequently, we identified a substantial number of potentially pleiotropic genes associated with NAFLD and sarcopenia using PLACO pleiotropic association analysis. Furthermore, a comprehensive MR analysis provided robust evidence supporting a causal relationship between NAFLD and sarcopenia. These findings provide a comprehensive perspective on the potential pathogenesis of sarcopenia and may lead to the development of novel treatment strategies.

The presence of a positive association between NAFLD and sarcopenia was established by a comprehensive Mendelian randomization analysis (MR), which is consistent with previous cross-sectional studies^25–28^. For instance, a prospective study of 225 Caucasian individuals demonstrated a linear increase in the prevalence of sarcopenia with the severity of liver fibrosis (OR = 2.36, P = 0.01). Furthermore, even after adjustment for confounding factors, sarcopenia was correlated with the severity of hepatic steatosis (OR = 2.02, P = 0.03)^29^. Consistent results have also been reported with invasive diagnostic methods. In a study by Koo et al.^8^, involving 309 samples with available liver histology, sarcopenia is nearly twice as common in individuals with NAFLD. Specifically, from 9% in the control group, prevalence rose to 18% in those with isolated fatty liver and then to 35% in those with NASH^30^. In addition, there is a double risk of developing NASH and significant fibrosis in patients with co-existing NAFLD and sarcopenia. When NAFLD and sarcopenia occur together, they may pose the highest risk for all-cause mortality and cardiovascular mortality. However, it is important to note that NAFLD and sarcopenia have an interactive effect on mortality rather than a cumulative effect^30^.

The relationship between NAFLD and sarcopenia involves a complex pathophysiological mechanism that has not been fully elucidated by researchers. Therefore, in this study, we explore the possible causes of the link between sarcopenia and NAFLD, including insulin resistance, inflammation, vitamin D deficiency, etc. The liver and skeletal muscle are both target organs for insulin. Insulin resistance, characterized by decreased responsiveness to insulin, can have detrimental effects on skeletal muscle. It promotes increased skeletal muscle breakdown, resulting in reduced mitochondrial content, impaired mitochondrial function, and decreased oxidative capacity. These factors contribute to the development of sarcopenia^31^. In addition, stimulation of fatty acid oxidation by hepatocytes leads to increased production of oxygen radicals. This process leads to lipid peroxidation and triggers the synthesis of pro-inflammatory cytokines. As a result, muscle protein breakdown is increased, ultimately contributing to the development of sarcopenia^32^. One study has demonstrated that tumor necrosis factor-alpha (TNF-α) is synthesized by the fibrotic liver and can be transported via systemic circulation to skeletal muscle, where it induces muscle atrophy^32^. Certain cytokines, such as fibroblast growth factor-21 (FGF-21), may also constitute a direct link between NAFLD and sarcopenia^33^. FGF-21 is predominantly synthesized by hepatocytes and plays a role in promoting glucose and lipid excretion from the body while enhancing mitochondrial function. Impairment of FGF-21 signaling may lead to reduced expression of PPAR γ-coactivator-1α, potentially contributing to the development of sarcopenia^34^. The mechanisms between NAFLD and sarcopenia are multifaceted and complex and go beyond the above description. Therefore, extensive and high-quality research is needed to further explore these mechanisms.

Our study further revealed that the pleiotropic genes obtained by PLACO analysis were enriched in pancreas, liver, heart, blood, brain and muscle tissue. The results of this enrichment analysis provide further evidence that insulin resistance may play an important role in NAFLD and sarcopenia. In compensatory hyperinsulinemia induced by insulin resistance, impaired inhibition of gluconeogenesis promotes protein hydrolysis and reduces protein synthesis^35^. The pituitary gland is responsible for releasing growth hormone (GH), which acts on the liver to stimulate the production of insulin-like growth factor-1 (IGF-1). IGF-1 is a crucial factor in brain neurogenesis and cognitive function, indicating that IGF-1 signaling may play a significant role in the communication between skeletal muscle and the brain^36^. Furthermore, studies suggest a potential association between impairment of the GH/IGF-1 axis and the risk of developing sarcopenic obesity, as well as the accumulation of ectopic fat in the liver^37,38^. Through enrichment analysis, the pathological mechanisms of NAFLD and sarcopenia are once again demonstrated to be the combined result of multiple factors and multi-organ involvement.

Our research holds significant importance both statistically and scientifically. First, genes are widely recognized as biologically significant functional units within an organism. Our analysis focuses on gene-centric analysis rather than individual SNPs. This approach allows us to capture the broader genetic landscape and provide a more comprehensive understanding of the relationship between NAFLD and sarcopenia. Secondly, we employ the PLACO method to detect gene pleiotropy. PLACO has been demonstrated to exhibit superior error accuracy and greater power performance compared to other analytical methods. By leveraging the strengths of PLACO, we enhance the reliability and robustness of our findings, contributing to the validity of our research outcomes. Thirdly, we identify and present a set of pleiotropic genes associated with both NAFLD and sarcopenia. These genes serve as valuable candidates for subsequent investigations and functional studies, allowing researchers to delve deeper into the shared genetic components and pathways involved in the development of NAFLD and sarcopenia. Fourth, our study conducts comprehensive enrichment analyses of NAFLD and sarcopenia, providing a foundation for future research on their pathogenesis. By uncovering the enriched biological processes, cellular components, and molecular functions, we offer valuable insights into the potential mechanisms driving the development and progression of these conditions.

However, there are some limitations to this study. However, it is important to acknowledge the limitations of this study. Firstly, the functional roles of the identified pleiotropic genes remain unclear. Further experimental studies are warranted to elucidate the specific mechanisms by which these genes contribute to the development of NAFLD and sarcopenia. Secondly, it is worth noting that our GWAS study focused exclusively on European populations. Therefore, the generalizability of our findings to other ethnic groups remains uncertain. Replication studies involving diverse populations are needed to assess the robustness and applicability of our results across different ethnicities. Thirdly, due to the unavailability of individual-level GWAS data, we were unable to stratify our study of NAFLD and sarcopenia by age and sex. This limitation hinders our ability to explore potential age- and sex-specific effects on the observed associations. Then, due to the fact that NAFLD patients refer to patients with one of the following ICD codes: ICD-10 K75.8 represents "non-alcoholic fatty liver disease", K76.0 represents "non-alcoholic fatty liver disease", or ICD-9 571.5 represents "non-alcoholic fatty liver disease". Therefore, it is highly likely that some of the 373,227 controls in this study also had some degree of NAFLD. Finally, due to data permission issues, we were unable to obtain corresponding individual information. Therefore, in this study, we mainly evaluated the relationship between NFALD and sarcopenia using the above indicators. However, considering the possibility of diagnostic bias in the healthy group, a large amount of research is still needed to further confirm the relationship between the two. Based on this, a large amount of research is still needed to further confirm the relationship between the two.

## Conclusion

In conclusion, our study employing comprehensive and novel statistical genetic bioinformatics approaches has revealed a genetic association between NAFLD and sarcopenia. Furthermore, we have identified a causal relationship between NAFLD and an increased risk of sarcopenia. These findings offer valuable insights into the genetic mechanisms underlying NAFLD and sarcopenia. Additionally, the identification of pleiotropic genes provides potential targets for future clinical drug therapies aimed at managing or treating NAFLD and sarcopenia.

### Supplementary Information

Supplementary Information 1.

Supplementary Information 2.

## Data Availability

The datasets used and analysed during the current study available from the corresponding author on reasonable request.
