# Supplementary figures and images for: Effects of nonalcoholic fatty liver disease on sarcopenia: evidence from genetic methods
Source: Sci Rep. 2024 Feb 1;14:2709. doi: 10.1038/s41598-024-53112-1 (PMC10834579; doi:10.1038/s41598-024-53112-1)

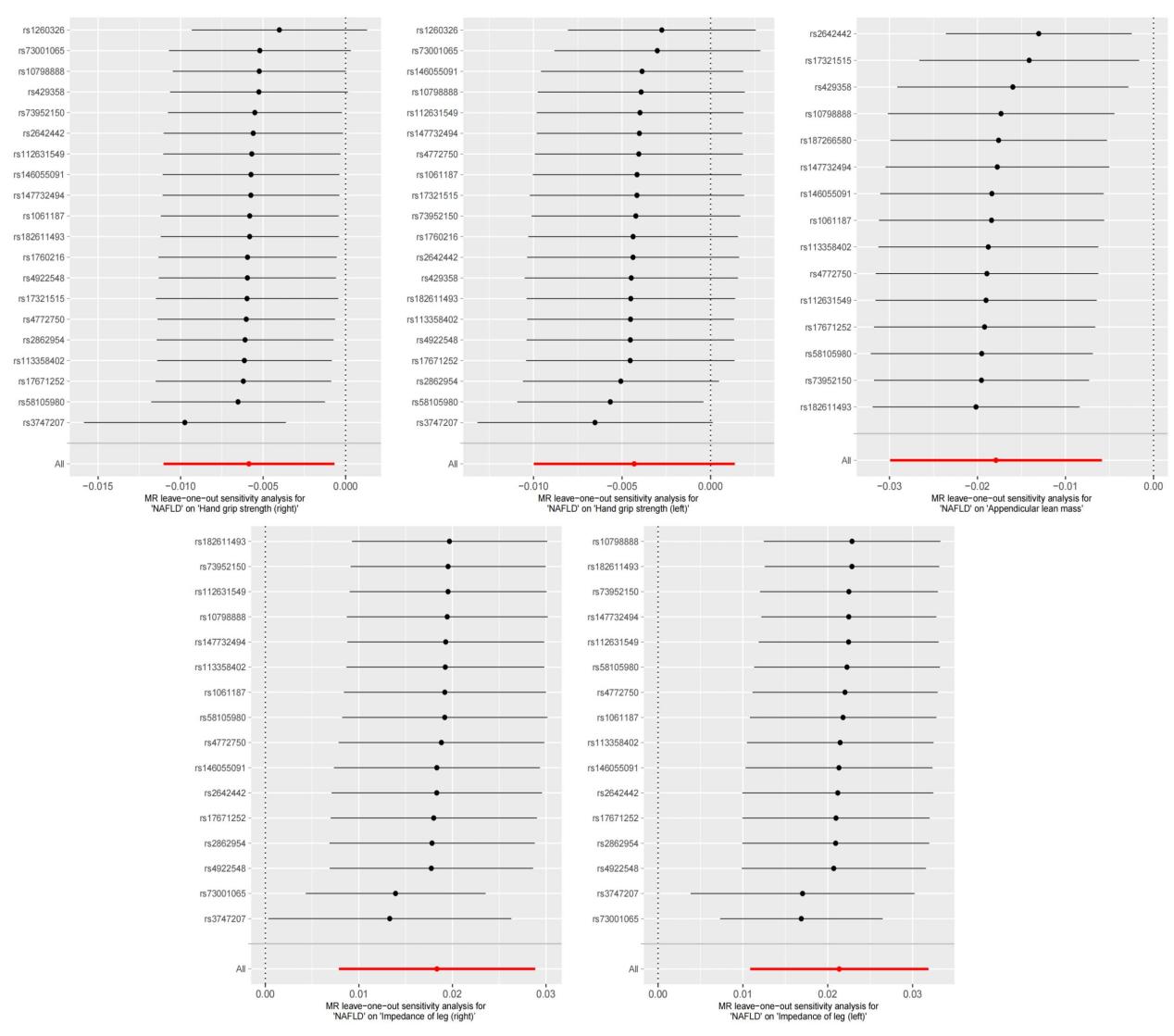


**Figure S1** LOO plot to visualize causal effect of NAFLD on sarcopenia.

Supplement: Supplementary file 1 — Supplementary Information 1. [file 41598_2024_53112_MOESM1_ESM.docx]
